# Associations between the cortisol awakening response and patient-evaluated stress and mood instability in patients with bipolar disorder: an exploratory study

**DOI:** 10.1186/s40345-020-00214-0

**Published:** 2021-03-01

**Authors:** Maria Faurholt-Jepsen, Vibe Gedsø Frøkjær, Arafat Nasser, Niklas Rye Jørgensen, Lars Vedel Kessing, Maj Vinberg

**Affiliations:** 1 Copenhagen Affective Disorder Research Center (CADIC), Psychiatric Center Copenhagen, Blegdamsvej 9, Rigshospitalet, 2100 Copenhagen, Denmark; 2 Faculty of Health and Medical Sciences, University of Copenhagen, Copenhagen, Denmark; 3 Neurobiology Research Unit, Copenhagen University Hospital, Rigshospitalet, Denmark; 4 Department of Clinical Biochemistry, Rigshospitalet, Copenhagen University Hospital, Copenhagen, Denmark; 5 Psychiatric Research Unit, Psychiatric Centre North Zealand, Faculty of Health and Medical Sciences, University of Copenhagen, Hillerød, Denmark

**Keywords:** Bipolar disorder, Cortisol awakening response, Smartphone

## Abstract

**Objective:**

The Cortisol Awakening Response (CAR) measured as the transient increase in cortisol levels following morning awakening appears to be a distinct feature of the HPA axis. Patients with bipolar disorder (BD) experience daily stress, mood instability (MI) and studies have shown disrupted HPA-axis dynamics. Aims: to evaluate (1) patient-evaluated stress against the CAR, (2) associations between the CAR and mood symptoms, and (3) the effect of smartphone-based treatment on the CAR.

**Methods:**

Patients with BD (n = 67) were randomized to the use of daily smartphone-based monitoring (the intervention group) or to the control group for six months. Clinically rated symptoms according to the Hamilton Depression Rating Scale 17-items (HDRS), the Young Mania Rating Scale (YMRS), patient-evaluated perceived stress using Cohen’s Perceived Stress Scale (PSS) and salivary awakening cortisol samples used for measuring the CAR were collected at baseline, after three and six months. In the intervention group, smartphone-based data on stress and MI were rated daily during the entire study period.

**Results:**

Smartphone-based patient-evaluated stress (*B: 134.14, 95% CI: 1.35; 266.92, p* = *0.048*) and MI (*B: 430.23, 95% CI: 52.41; 808.04, p* = *0.026*) mapped onto increased CAR. No statistically significant associations between the CAR and patient-evaluated PSS or the HDRS and the YMRS, respectively were found. There was no statistically significant effect of smartphone-based treatment on the CAR.

**Conclusion:**

Our data, of preliminary character, found smartphone-based patient-evaluations of stress and mood instability as read outs that reflect CAR dynamics. Smartphone-supported clinical care did not in itself appear to disturb CAR dynamics.

## Introduction

The Cortisol Awakening Response (CAR) measured as the transient increase in cortisol levels following morning awakening appears to be a distinct feature of the hypothalamus–pituitary–adrenal (HPA) axis, is associated with a wide range of variables reflecting health status and differs according to age and sex (Fries et al. 2009; Larsson et al. 2009; Stalder et al. 2011; Roelfsema 2017). A meta-analysis including forty-one studies and two individual studies showed that bipolar disorder was associated with increased basal levels of cortisol and decreased inhibitory feedback capacity (i.e. increased cortisol post-dexamethasone), and that case–control differences in cortisol levels were positively associated with mania (Jabben 2011; Belvederi Murri et al. 2016; Huang 2017). Familial risk for mood disorders is associated with a compromised inhibitory feedback regulation of the HPA-axis (Modell 1998), which is a significant predictor for relapse of depressive episodes in patients with a history of depression (Zobel 2001; Vrshek-Schallhorn 2013). Overall, previous studies in patients with affective disorders have thus shown changed cortisol patterns during depressive episodes and manic episodes and while in remission. Patients with bipolar disorder further exhibit vulnerability to stress, which in addition to a hyperactive HPA-axis seem to play a key role in the pathophysiology of the disorder (Belvederi Murri et al. 2016; Cervantes et al. 2001; Fries 2014; Coello et al. 2018). Patients with bipolar disorder often report an increased sensitivity to experiencing distress, which is interconnected to mood instability (MI) (Faurholt-Jepsen 2019b; Stanislaus 2020). Further, a substantial proportion of patients with bipolar disorder experience daily subsyndromal mood swings, and MI reflects the amount and degree of mood swings. MI is further associated with poor prognosis including impaired functioning and quality of life, increased perceived stress, increased risk of hospitalization, and a higher risk of relapse (Faurholt-Jepsen 2019b; Bopp 2010; Strejilevich 2013; Patel 2015; Gershon and Eidelman 2015; O’Donnell 2018). Due to the increased focus on the impact of mood instability, mood instability has been suggested as an independent new treatment target and as a more sensitive measure of outcome in randomized controlled trials (RCTs) than for example relapse or recurrence of affective episodes (Bopp 2010; Bonsall et al. 2012; Saunders 2016; Broome et al. 2015). However, previous studies measuring mood instability have included data based on weekly assessments, and data were often collected using paper-based methods thereby increasing the risk of patient recall bias (Strejilevich 2013).

The rapid evolution and ubiquity of mobile networks have resulted in the increasing development tools for remote and real-time self-monitoring (Lal and Adair 2014). Real-time self-monitoring of momentary or very recent experiences have important advantages over retrospective reports, as they contain less bias and allow for unobtrusive monitoring during naturalistic settings (Stone et al. 2003). Studies using daily assessments have found associations between increased CAR and for example chronic stress in daily life and reduced distress responses to daily life stress (Schlotz et al. 2004; Powell and Schlotz 2012; Schlotz 2019). The authors have previously developed and investigated the usability, validity and effect of a smartphone-based self-monitoring system (the previous MONARCA system) in patients with bipolar disorder (Faurholt-Jepsen 2015a, 2015b,2019a). The MONARCA system offers a unique possibility to obtain detailed data on how patients with bipolar disorder experience daily hassles, and in the previous studies patients with bipolar disorders report higher levels of daily stress than healthy control individuals (Faurholt-Jepsen 2019b; Stanislaus 2020). However, it has not previously been investigated whether smartphone-based daily patient-evaluated stress align with aspects of biological stress hormone reactivity, i.e. HPA-axis dynamics in terms of CAR.

Here we report on the tertiary outcome measures collected as part of a randomized controlled trial (RCT) that investigated the effect of a smartphone-based monitoring system on the severity of depressive and manic symptoms in patients with bipolar disorder (the MONARCA I trial) (Faurholt-Jepsen 2014, 2013). In summary, overall the MONARCA I trial found no differences in the severity of depressive and manic symptoms (primary outcome) measured using the Hamilton Depression Rating Scale 17-items (HDRS) (Hamilton 1967) and the Young Mania Rating Scale (YMRS) (Young et al. 1978), respectively, between the intervention group and the control group (Faurholt-Jepsen 2015a). However, sub-group analyses suggested that the smartphone-based monitoring system may sustain the level of depressive symptoms and reduce the level of manic symptoms and thereby affect the level of CAR.

### Aims and hypotheses

In patients with bipolar disorder the present study, of preliminary character, aimed to (1) evaluate the association between perceived stress measured using Cohen’s perceived stress scale (PSS) (Cohen et al. 1983) and daily smartphone-based measures of stress against the CAR; (2) investigate the association between the CAR and clinically evaluated mood symptoms (measured using the HDRS, the YMRS) and patient-evaluated mood instability (MI) measured using daily smartphone data, respectively, and (3) investigate the effect of smartphone-based treatment on the level of the CAR.

We hypothesized, that there would be a positive association between the CAR and daily patient-evaluated stress and MI, respectively. Further, we hypothesized, that there would be a positive association between the CAR and clinical observed mood symptoms according to the HDRS and the YMRS, respectively, and that smartphone-based treatment could reduce the CAR.

## Material and methods

### Design, settings and participants

The present study presents data collected as part of a RCT investigating the effect of smartphone-based monitoring in patients with bipolar disorder (the MONARCA I trial) (Faurholt-Jepsen 2015a).

#### The MONARCA I trial

The patients were recruited from the Copenhagen Clinic for Affective Disorders, Copenhagen, Denmark during a period from September 2011 to March 2013. Patients were invited to participate in the trial following referral to the clinic and were randomized 1:1 to using a smartphone-based monitoring system (the MONARCA system) for daily electronic self-monitoring (the intervention group) (further description below) or to the use of a smartphone without the monitoring system for normal communicative purposes with no daily electronic self-monitoring (the control group). Regardless the allocation group, patients were followed for six months. The inclusion criteria were a bipolar disorder diagnosis according to ICD-10 and DSM-IV using the Schedules for Clinical Assessment in Neuropsychiatry (SCAN) interview (Wing et al. 1990) (conducted by an experienced medical doctor and researcher, MFJ), age between 18 to 60 years, a Hamilton Depression Rating Scale 17-item (HDRS-17) score ≤ 17 (Hamilton 1967) and a Young Mania Rating Scale (YMRS) score ≤ 17 (Young et al. 1978) at the time of inclusion. The exclusion criteria were pregnancy, a lack of Danish language skills, inability to learn the technicalities for using a smartphone, unwilling to use the trial smartphone as the primary cell phone, and severely physical illness or schizophrenia, schizotypal or delusional disorders according to the SCAN interview.

A total of 78 patients with bipolar disorder receiving treatment at the Copenhagen Clinic for Affective Disorder, Denmark at the time of the study were included. Among the 78 patients a total of 67 patients showed up for clinical assessments and were included in the analyses.

### Clinical assessments and questionnaires

During the entire trial period of six months, monthly clinical ratings were conducted face-to-face by an MD and trained researcher (MFJ) and questionnaires were collected.

Clinical ratings: The severity of depressive and manic symptoms for the last four days was evaluated using the HDRS (Hamilton 1967) and the YMRS (Young et al. 1978), respectively. Data on functioning were clinically rated using the Functional Assessment Short Test (FAST), which is a 24-item interviewer-administrated interview concerning autonomy, occupational functioning, cognitive functioning, financial issues, interpersonal relationships and leisure time (Rosa 2007); scores above 11 indicate impaired functioning. All clinical ratings were conducted by a clinician (MFJ) who was blinded to the patients’ group of allocation during the entire MONARCA I trial, and thus the clinical ratings were blinded.

Patient-based questionnaires: The severity of perceived stress was evaluated by the patients using Cohen’s Perceived Stress Scale, which is a 14-item questionnaire measuring the degree to which situations in one’s life are appraised as stressful reflecting the last 14 days. Higher scores indicate higher perceived stress (Cohen et al. 1983).

### Patient-reported daily monitoring via smartphones

In brief, the MONARCA system consisted of the following: Patients allocated to the intervention group of the MONARCA I trial were provided with an Android smartphone with the MONARCA system installed. At a self-chosen time point on a daily basis during the six months trial period, patients in the intervention group were reminded by an alarm and prompted to use the smartphone-based system for self-assessment of mental distress experienced during that particular day as a whole (Faurholt-Jepsen 2014). The app allowed for anchored daily self-assessment of stress (scored on a scale from 0, 1, 2, 3, 4, 5) and mood (scored on a scale from − 3 to + 3) (and other items such as sleep, medication etc.). The intervention also included a clinical feed-back loop to a study nurse, who went through the smartphone-based data on a daily basis and contacted the patients in case of signs of deterioration. Further details regarding the MONARCA system and the intervention during the MONARCA trial have been described elsewhere (Faurholt-Jepsen 2014, 2013). Patients allocated to the control group did not provide any self-assessed smartphone data during the trial.

### Cortisol Awakening Response

Regardless the group of allocation in the MONARCA trial, Cortisol Awakening Response (CAR) samples were collected at baseline, after three months and six months on all patients. The CAR is based on five serial measurements of salivary cortisol over the first hour from awakening (at 0, 15, 30, 45 and 60 min. after awakening) and computed as the area under the curve with respect to increase from baseline level at awakening (AUC_i_) controlled for the level of salivary cortisol in sample 1. Patient instructions, home sampling procedures, and cortisol analyses were carried out as described elsewhere (Frokjaer 2013). At each visit, patients returned a paper with handwritten registered timings of collected saliva samples, and it was checked by the researchers that the timings complied with those specified in the home sampling procedures. All samples were collected at the participant’s home and were returned at the three assessment days and kept frozen at − 80 °C until assayed in one single batch. Salivary cortisol concentrations were determined by a chemiluminescence immunoassay (CLIA) method on the IDS-iSYS automatic analyzer (IDS PLC, Boldon, UK). The intra- and inter-assay variation was up < 15%, which adhered to the standards of the lab. However, if this variation had been smaller, we might have detected more robust results.

### Statistical methods

Descriptive analyses were produced using percentages, medians, interquartile range, means and standard deviations. In one case the 60 min. a salivary cortisol sample was missing, and the CAR was calculated using the available four salivary samples in order to calculate the AUC_i_. All statistical analyses including co-variates were defined a priori and computed using linear mixed effect regression models. These regression models allow for both *within-*individual and *between-*individual variations over time with repeated measurements of the variables considered in the analyses. Thus, for each of the analyses we employed a two-leveled linear mixed regression model with the first level representing the repeated measurements per patient of the variables of interest *within-*individuals and the second level representing the *between-*individuals variation of the variables of interest. All models included a fixed effect of visit number (baseline, three-months, six-months) and a participant specific random intercept and a participant specific random effect to account for patient specific correlations between the dependent variables (CAR) and the independent variables (e.g.: PSS, HDRS, YMRS) over time due to repeated measurements. All clinical ratings and questionnaire data from baseline, three-months and six-month) were included corresponding to the CAR at that time point in the analyses. In the linear mixed effect regression models, analyses were conducted assuming that missing data was missing at random. These models will use the full available data set. Thus, we assumed data was missing at random and analyzed data to get an unbiased estimate.

Other covariates were specified as fixed effects in the models. For all analyses, models were adjusted for age and gender, as these covariates may affect the level of the CAR (Larsson et al. 2009; Roelfsema 2017).

Concerning aim 1: to investigate the association between daily smartphone-based measures of patient-evaluated stress collected from patients in the intervention group using the MONARCA system coupling to the CAR, all daily smartphone-based data on stress available from all days during the entire trial period of six months was used in the analyses. There is no consensus on how to report the performance and variance in hierarchical linear models. In the present paper, we reported the Snijders and Bosker’s method (Snijders and Bosker 1994), which can be interpreted as the proportion of variance in math achievement explained by the covariates. Values of 0.02 indicate a small proportion of variance explained by the covariates, 0.15 a proportion, and 0.35 a large proportion.

Concerning aim 2: to investigate the association between MI measured using daily smartphone-based data collected from patients in the intervention group using the MONARCA system and the CAR, measures of mood from all days during the entire trial period were used in the analyses. MI was operationalized and calculated as the number of mood changes, on a scale from − 3 to + 3, from day to day divided by the number of weeks followed between baseline to three months and from three months to six months. This method has been used previously by the authors and others (Strejilevich 2013; Faurholt-Jepsen 2015). Thus, the MI measure reflects the number of changes in patient-based mood evaluations, even at a subclinical level, rather than the extent of mood changes.

Concerning aim 3: to investigate the effect of smartphone-based treatment on the CAR, difference in CAR between the two groups (intervention group and control group) during the entire trial period of six months was investigated. The effect of interaction between visit number and randomization group on the CAR was investigated, but not reported (*p* = 0.48).

Model assumptions concerning deviations from normality and heteroscedasticity were checked visually by means of residual plots, and QQ plots for each of the statistical analyses. Since this is the first study to investigate the association between perceived stress measured using smartphones and the CAR, we were not able to conduct statistical power calculations. The variance inflation factor (VIF) identifying the correlation between independent variables and the strength of that correlation was calculated to be < 5, and therefore not severe enough to warrant corrective measures. The statistical significance (two-tailed) limit was set at *p* ≤ 0.05. Data were entered using Excel and STATA version 13 (StataCorp, LP, College Station, TX, USA) was used for statistical analyses.

### Ethical considerations

The trial was approved by the Regional Ethics Committee of the Capital Region of Denmark (H-2-2011-056) and the Danish Data protection agency (2013-41-1710). The law regarding the handling of personal data was respected. Prior to commencement the trials were registered at ClinicalTrials.gov (NCT01446406). The electronic data collected from the smartphones were stored at a secure server at Concern IT, Capital Region, Denmark (I-suite number RHP-292 2011-03). The trial complied with the Helsinki Declaration of 1975, as revised in 2008.

## Results

### Background characteristics

Of the 67 included patient, 33 patients allocated to the intervention group providing daily monitoring of stress and mood via smartphones were included in the present study of analyses concerning only smartphone-based self-monitoring. During the trial period patients randomized to the intervention group provided daily mood monitoring 94.5% of the days and daily stress monitoring 88.6% of the days. The patients had a median stress level of 1 [IQR 0–2] and a median mood level of 0 [IQR 0–1] measured using the smartphone-based system. Further, 34 patients were allocated to the control group and did not provide smartphone-based data, but they were included in the analyses concerning clinical face-to face ratings and PSS data. Two patients, one in the intervention group (due to travelling abroad) and one in the control group (due to non-response), had a discontinued intervention after three months follow-up. Only few patients’ visits were missing (3.6% in the intervention group and 3.8% in the control group).

Details on background characteristics and clinical information are presented in Table 1.

**Table 1:** Background characteristics and clinical information of study participants, N = 67

	Intervention group (n = 33)	Control group (n = 34)
Age, years	29.1 (7.5)	29.5 (9.4)
Female sex, % (n)	65.7 (22)	68.6 (23)
Clinical history		
BPI diagnosis^a^, % (n)	60.0 (20)	74.3 (25)
Previous psychiatric admissions, number	1 [1, 2]	1 [1, 2]
Previous depressive episodes, number	4 [2–10]	4 [2–5]
Previous manic episodes, number	3 [2–6]	2 [1–5]
HDRS-17 score during the study	8 [4–14]	8 [4–14]
YMRS score during the study	2 [0–4]	2 [0–5]
PSS score during the study	18.5 (9.0)	18.9 (9.2)
FAST score during the study	22.1 (16.7)	23.7 (16.5)
Mood measured daily using smartphones^b^	− 0.32 (0.73)	–
S tress measured daily using smartphone^c^	0.93 (1.1)	–

Data are mean (SD), %(n) or median [IQR] unless otherwise stated; ^a^Bipolar disorder type I disorder; *HDRS-17* The Hamilton depression rating scale 17 items (median of 7 monthly ratings); *YMRS* The Young mania rating scale (median of 7seven monthly ratings); *PSS *Cohen’s Percieved Stress Scale (mean of 7 monthly ratings); *FAST *The Functioning Assessment Short Scale (mean of 7 monthly ratings); ^b^Mood measured daily using smartphone was evaluated on a scale from − 3 to + 3; ^c^Stress measured daily using smartphone was evaluated on a scale from 0 to 5

A total of 116 samples were excluded due to inadequate saliva volume. Thus, a total of 951 salivary cortisol samples, collected between 6 a.m. and 10 a.m., were available for statistical analyses. None of the samples were excluded due to invalid timing. A total of 79% of the patients provided all three CAR samples during the trial period. Details concerning cortisol levels are presented in Table 2.

**Table 2:** Salivary cortisol measures in patients with bipolar disorder during a 6 months study period, N = 67

Number of salivary samples, total	951
Raw concentration of cortisol (nmol/l) in awakening sample, baseline	11.11 (9.94)
Raw concentration of cortisol (nmol/l) in awakening sample, three months	11.26 (9.55)
Raw concentration of cortisol (nmol/l) in awakening sample, six months	10.08 (5.67)
Area under the curve with respect to increase (nmol/l), baseline	91.23 (545.07)
Area under the curve with respect to increase (nmol/l), three months	186.70 (350.20)
Area under the curve with respect to increase (nmol/l), six months	297.45 (421.29)

Data are mean (SD) unless otherwise stated

The average raw area under the curve with respect to increase of cortisol (AUC_i_) at baseline was 91.23 (SD 545.07) nmol/l*min, at visit three months 186.70 (SD 350.20) nmol/l*min, and at six months 297.45 (SD 421.29) nmol/l*min. Raw concentrations of cortisol in the awakening sample are presented in Table 2.

### Association between the Cortisol Awakening Response and patient-evaluated stress

There was no statistically significant association between patient-evaluated *non-smart-phone-based* stress measured by the PSS and the CAR in the models adjusted for age and gender (*B: − 1.63, 95% CI: − 10.53; 7.26, p* = *0.72 (N* = *67)*) (Snijders and Boskers: 0.17) (Table 3). The estimates were not altered to a large extent when only including patients from the intervention group, and therefore not reported.

**Table 3:** Associations between cortisol awakening response (CAR) and patient-evaluated perceived stress and mood instability measured daily using smartphones, questionnaire-based perceived stress, clinically evaluated functioning, depressive symptoms and manic symptoms, respectively in patients with bipolar disorder

	Model^a^		
	B	95% CI	*p*
Analyses including all patients, n = 67			
CAR			
PSS ^c^	− 1.63	− 10.53; 7.26	0.72
FAST	− 1.18	− 6.48; 4.12	0.66
HDRS	− 6.79	− 17.89; 4.30	0.23
YMRS	− 12.88	− 29.92; 4.15	0.14
Analyses including only patients using a smartphone-based monitoring system, n = 33			
CAR			
Stress measured using smartphones^b^	134.14	1.35; 266.92	0.048
Mood Instability measured using smartphones^c^	430.23	52.41; 808.04	0.026
PSS	10.73	− 2.78; 24.23	0.12
FAST	3.72	− 5.64; 13.09	0.44
HDRS	4.97	− 12.36; 22.31	0.58
YMRS	− 3.93	− 27.46; 19.61	0.74
Effect of smartphone-based treatment. Control group serve as reference, n = 67			
CAR	− 16.57	− 183.11; 216.25	0.87

^a^Model: Adjusted for age and gender

*CAR* Cortisol Awakening Response; *PSS*: Perceived stress measured using Cohen’s Perceived Stress Scale; *FAST* Psychosocial functioning measured using Functional Assessment Short Test; *HDRS* Hamilton Depression Rating Scale; *YMRS* Young Mania Rating Scale; ^b^Stress measured using smartphone was evaluated on a scale from 0 to 5; ^c^Mood Instability calculated as the number of mood changes from day to day from baseline to three months and from three months to 6 months

**Fig. 1:**
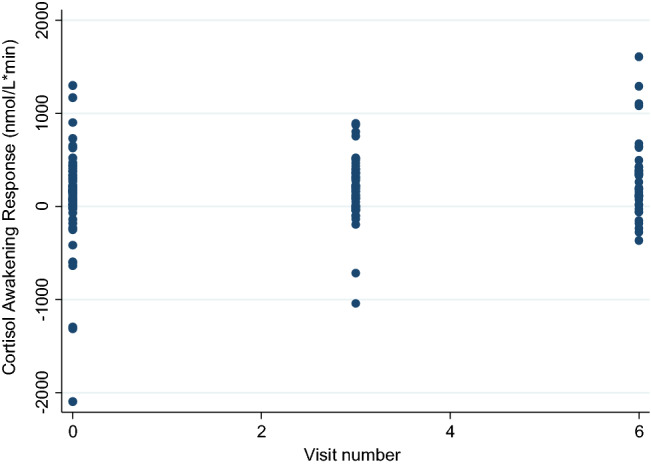
Scatter plot of CAR and smartphone-based monitoring of stress. Each dot represents multiple smartphone-based patient-reported evaluations of stress level and CAR measures

There was a statistically significant positive association between daily smartphone-based patient-evaluated stress and the CAR in the models adjusted for age and gender (*B: 134.14, 95% CI: 1.35; 266.92, p* = *0.048 (n* = *33*)) (Snijders and Boskers: 0.05). Thus, for every increase of 1 point on the smartphone-based stress measure there was an increase of 134.14 nmol/l*min of CAR (Table 3 and Fig. 1).

In addition, there was a statistically significant positive association between patient-evaluated stress measured using the PSS and patient-evaluated stress measured using smartphones in the models adjusted for age and gender (*B: 3.33, 95% CI: 2.02; 4.65, p* < *0.0001* (n = 33)).

Exploratory analyses including bipolar disorder subtype (bipolar disorder type I or II) as a covariate did not alter these estimates.

### Association between Cortisol Awakening Response and face-to-face clinician-evaluated depressive symptoms and manic symptoms, repsectively

The results from the adjusted linear mixed effect models regarding the association between the CAR and clinician-evaluated depressive and manic symptoms measured using the HDRS and the YMRS, respectively are presented in Table 3.

We saw no statistically significant associations between neither clinician-evaluated depressive symptoms according to the HDRS, nor manic symptoms according to the YMRS, and the CAR in the models adjusted for age and gender (HDRS: (*B: − 6.79, 95% CI: − 17.89; 4.30, p* = *0.23*), YMRS: (*B: − 12.88, 95% CI: − 29.92; 4.15, p* = *0.14*)).

### Association between Cortisol Awakening Response and patients evaluated smartphone-based mood instability

The results from the adjusted linear mixed effect models regarding the association between the CAR and smartphone-based MI, respectively are presented in Table 3.

We saw a statistically significant positive association between smartphone-based MI (based on daily smartphone-based data on mood) and the CAR in the models adjusted for age and gender (*B: 430.23, 95% CI: 52.41; 808.04, p* = *0.026*). Thus, for every increase of 1 point in MI there was an increase of 430.23 nmol/l*min of the CAR (Table 3).

When restricting the analyses to include data from patients using the smartphone-based monitoring system only (the intervention group) the estimates concerning associations between the CAR and clinician-evaluated depressive and manic symptoms measured using the HDRS and the YMRS, and smartphone-based MI, did not change substantially and were therefore not reported. Likewise, exploratory analyses including bipolar disorder subtype (bipolar disorder type I or II) as a covariate did not alter these estimates in the analyses.

### The effect of smartphone-based treatment on the levels of CAR

Difference in the CAR between the intervention group (patients using the smartphone-based MONARCA system) and the control group is presented in Table 3. There was no statistically significant difference in the magnitude of CAR between the intervention group and the control group (*B: − 16.57, 95% CI: − 183.11; 216.25, p* = *0.87*). Exploratory analyses including bipolar disorder subtype (bipolar disorder type I or II) as a covariate did not alter these estimates in the analyses. The effect of interaction between visit number and randomization group on the CAR was investigated but is not reported (*p* = 0.48).

## Discussion

For the first time, we here evaluated associations between biological measures of stress-hormone dynamics and high-resolution smartphone-based patient-evaluated daily levels of stress and MI and non-smartphone-based measures of perceived stress, and severity of depressive and manic symptoms in patients with bipolar disorder. Intriguingly, as hypothesized, the CAR was positively associated with daily patient reports of stress and MI measured via smartphones, but not with clinician-rated severity of depressive or manic symptoms or non-smartphone-based patient-reported stress. Also, smartphone-supported clinical care did not in itself reduce the CAR relative to non-smartphone-based care.

Interestingly, we found that increased perceived stress measured daily using smartphones, but not with questionnaire-based perceived stress, was associated with contemporary increased CAR levels. Thus, in contrast to questionnaire-based data and clinical interviews collected at low time-resolution, the fine-grained serial stress and MI measurements capture changes in the CAR. Fine-grained measurements may increase illness insight and improve patients' ability to register symptoms, and thereby facilitate the integrations of psychoeducational elements. Real-time self-monitoring of momentary or very recent experiences have important advantages over retrospective reports, as they contain less bias and allow for unobtrusive monitoring during naturalistic settings (Stone et al. 2003). Other studies in different groups of patients using daily assessments have found associations between increased CAR and for example chronic stress in daily life and reduced distress responses to daily life stress (Schlotz et al. 2004; Powell and Schlotz 2012; Schlotz 2019). However, if the included patients with bipolar disorder had been in a later more progressed stage of their illness it may have translated into more disrupted HPA-axis dynamics expressed as a blunted CAR response (Huang 2017). Nevertheless, the present sample consisted of relatively young patients (mean age 29.4 years), who had relatively low number of previous affective episodes and thus may be in an earlier stage of the disorder that other studies, which seem to be reflected in the present finding of an increased HPA-axis reactivity pattern. So, the HPA axis may express hyper-reactivity in the early stages but could change over the course of illness in line with the kindling hypotheses, in which psychosocial stressors are thought to play a greater role in the initial affective episodes than in subsequent affective episodes (Post 1992). According to this hypothesis, stressors may leave a long-term vulnerability, i.e. a scarring effect. This leads to a lowering of the threshold of the stress exposure required for a new affective episode, so that, over time, relatively minor stressors may trigger new affective episodes (Post 1992). Overall, recent adversities measured as subjective stress seem to impact the patients everyday living as reflected by higher level of MI, which potentially reflects a hypersensitivity to stress. However, in the present study, it is a limitation that we did not measure early adversities e.g. childhood trauma early experiences probably impact vulnerability to developing depressive episodes in response to future stressors (Havermans et al. 2011; Shapero 2017). Finally, whether the kindling hypothesis translates into biological stress measures e.g. the CAR is not possible to reveal from the present study, and there is a lack of longitudinal studies including repeated biological measures of HPA axis dynamics in bipolar disorder.

### Strengths of the present study

The included patients were clinically well characterized and were receiving treatment in a specialized mood disorder clinic. Regarding the smartphone-based monitoring system, the MONARCA system has previously been shown to be easy to use and to have a high usability (Bardram et al. 2013). The clinical assessments used for the patients included the HDRS and the YMRS, which, in contrast to the questionnaires, are clinical face to face rating scales administered by an experienced clinical researcher (MFJ) who was blinded to all smartphone and the CAR data during the study. Previous studies on the use of the MONARCA system showed that patients were able to validly evaluate their mood as compared with clinically rated severity of depressive and manic symptoms according to the HDRS and YMRS, respectively (Faurholt-Jepsen 2015b). Finally, all CAR samples were analyzed in one single batch eliminating any measurement variation between batches.

### Limitations

Limitations to the present study of preliminary character should be mentioned. First, regarding the scale used for smartphone-based monitoring of stress, the severity of stress was assessed using a scale from 0 to 5, which limited the opportunity to evaluate associations using a scale with higher granularity. Thus, minor symptoms of stress could have been both under- and over-reported by the patients, affecting the associations presented in the present study. Second, the findings were based on the analyses of associations between variables, and thus any potential causality between the measures cannot be evaluated from the present study. Although the included covariates did not alter the estimates to a large extent, other possible confounding factors such as psychopharmacological treatment that were not included in the statistical analyses could have affected the associations, and thus any interpretation should be made with caution. Third, the majority of patients screened for participation in the MONARCA I trial declined to participate for various reasons (Faurholt-Jepsen 2015a). Thus, one might speculate if patients who were favourably oriented towards a technology-based remote monitoring programme may have differed from patients who were not, and our results may not generalize to all segments of a bipolar population. Fourth, we did not have access to adjacent smartphone-based stress measures and CAR in healthy individuals or individuals at high risk for bipolar disorder. Thus, we cannot determine if our findings are specific to patients with bipolar disorder. We collected the CAR samples three times during the present study, and therefore there is a lack of correspondence in time between the CAR samples and the daily smartphone responses. Any potential changes in CAR between the three times where the CAR samples were collected were therefore not available. The association between the CAR and the daily smartphone responses therefore does not reflect this., the use of smartphone-based technology could have been assessed in combination with e.g. actigraphy or a "Fitbit"-like item, which could systematically collect information on energy and activity in more details. Sixth, to date there is no clear consensus on the definition, measurement, use and reporting of MI in patients with bipolar disorder, in the present report we used a combination of measures done in previous studies (Faurholt-Jepsen 2019b, 2015; Strejilevich 2013; O’Donnell 2018; Ebner-Priemer et al. 2009). Using, defining, measuring and reporting MI in a standardized way across studies could improve the quality, reproducibility and comparison of results between studies. Seventh, patients included in the present study were recruited as part of a larger RCT investigating the effect of smartphone-based monitoring. Thus, the sample size and follow up period were defined according to the RCT’s design. It is possible that a larger sample with a longer follow up period could have resulted in other findings. Furthermore, the findings that smartphone-based monitoring did not reduce the level of CAR may be due to low power. Studies investigating the effect of smartphone-based monitoring on CAR should include a larger sample and follow patient for longer time to allow for changes in these measures. Since the findings from the present study are hypotheses generating, we did not account for multiple testing in the present study, thus we interpret our results with caution. Finally, as mentioned we did not have access to data on childhood trauma. Information regarding early adverse experiences as childhood trauma would have been of interest to include as a covariate in the analyses, as childhood trauma seems to contribute to alterations of affect regulation, impulse control, and cognitive functioning which might decrease the ability to cope with later stressors (Aas 2016).

### Conclusions

Our data, from the present study of preliminary character, found smartphone-based patient-evaluations of stress and mood instability as read outs that reflect the CAR dynamics. Smartphone-supported clinical care did not in itself appear to reduce the CAR dynamics.

## Data Availability

Not applicable.
